# Effect of Fiber Characteristics on the Structure and Properties of Quartz Fiber Felt Reinforced Silica-Polybenzoxazine Aerogel Composites

**DOI:** 10.3390/gels10100613

**Published:** 2024-09-24

**Authors:** Lanfang Liu, Liangjun Li, Yijie Hu, Junzong Feng, Yonggang Jiang, Jian Feng

**Affiliations:** Science and Technology on Advanced Ceramic Fibers and Composites Laboratory, College of Aerospace Science and Engineering, National University of Defense Technology, Changsha 410073, China; liulanfang421@163.com (L.L.); hyjscut2013@163.com (Y.H.); junzongfeng@nudt.edu.cn (J.F.); jygemail@nudt.edu.cn (Y.J.)

**Keywords:** quartz fiber felt reinforced silica-polybenzoxazine aerogel, fiber diameter, thermal insulation performance, high temperature, mechanical property

## Abstract

Fiber-reinforced aerogel composites are widely used for thermal protection. The properties of the fibers play a critical role in determining the structure and properties of the final aerogel composite. However, the effects of the fiber’s characteristics on the structure and properties of the aerogel composite have rarely been studied. Herein, we prepared quartz fiber felt-reinforced silica-polybenzoxazine aerogel composite (QF/PBSAs) with different fiber diameters using a simple copolymerization process with the ambient pressure drying method. The reasons for the effects of fiber diameter on the structure and properties of the aerogel composites were investigated. The results showed that the pore structure of the aerogel composites was affected by the fiber diameter, which led to significant changes in the mechanical behavior and thermal insulation performance. At room temperature, pore structure and density were found to be the main factors influencing the thermal conductivity of the composites. At elevated temperatures, the radiative thermal conductivity (λr) plays a dominant role, and reducing the fiber diameter suppressed λr, thus decreasing the thermal conductivity. When the QF/PBSAs were exposed to a 1200 °C butane flame, the PBS aerogel was pyrolyzed, and the pyrolysis gas carried away a large amount of heat and formed a thermal barrier in the interfacial layer, at which time λr and the pyrolysis of the PBS aerogel jointly determined the backside temperature of the composites. The results of this study can provide valuable guidance for the application of polybenzoxazine aerogel composites in the field of thermal protection.

## 1. Introduction

With the rapid development of aerospace technology, vehicle service environments are becoming increasingly complex and harsh [1,2,3]. To ensure safe flight and normal operation, thermal protection materials must withstand high-temperature airflow, aerodynamic loads, and long-term exposure to aerobic conditions. These demands present significant challenges for traditional materials [4,5,6]. Therefore, the development of lightweight, oxidation-resistant, high-efficiency thermal insulation, ablation-resistant and high-strength thermal protection materials is an urgent problem to be solved.

To meet the demand for thermal protection materials for aircraft, researchers have performed substantial research on this subject, with a particular focus on silicone-based and phenolic resin-based thermal protection materials [7,8,9]. Among these, the low-density carbon fiber-reinforced phenolic resin aerogel (LCF/PR) has attracted considerable interest due to its favorable low cost, low density, and exceptional ablative and thermal insulation properties [10,11]. However, the intrinsic poor thermal stability and oxidation resistance of carbon-based materials have resulted in a notable backward ablation when exposed to prolonged aerobic airflow [12,13]. This subsequently leads to an increased transfer of heat from the surface to the interior, which may potentially compromise flight safety and passenger well-being [14,15]. Hence, enhancing the thermal stability and mass residual of LCF/PR aerogel thermal protection materials is crucial to addressing these issues.

Generally, the thermal stability and mass residual of LCF/PR can be enhanced by two routes, namely, the introduction of inorganic components to modify the resin matrix or by replacing the carbon fiber with inorganic ceramic fibers that are resistant to high-temperature oxidation. Polybenzoxazine (PBO), as a nitrogen-containing phenolic resin, has received widespread attention in recent years due to its flexible designability of molecular structure, low cost and high residual carbon, and is expected to replace phenolic resins as a new generation of thermal protection materials [16,17,18]. Polybenzoxazine aerogels offer the benefits of PBO and aerogel, making them a promising choice for advanced thermal protection materials in aerospace [19]. However, the limited application of pure PBO aerogels in prolonged high-temperature conditions is attributed to their poor thermal stability and low mass residual in high-temperature aerobic environments [20]. Generally, the thermal stability and mass residual rate of organic aerogels were enhanced through the incorporation of inorganic components. In recent years, researchers have investigated the incorporation of various types of silicon, such as polysiloxane and silica, to improve the thermal stability and ablative properties of polybenzoxazine aerogels [21,22]. However, introducing a large amount of silicon into PBO aerogels often led to phase separation in the cured hybrid aerogels [23]. This was due to the asynchronous rates of phase separation and cross-linking between polybenzoxazine and various types of silicon during the gelation process, ultimately causing a decline in their properties [23,24]. Therefore, solving the problem of balancing the fine structure of a hybrid aerogel with a high mass residual is an effective way to improve the ablation resistance, oxidation resistance and high-efficiency thermal insulation of PBO aerogel composites. The silica-modified polybenzoxazine nanoporous aerogel (PBSA) with a core–shell structure developed by us exhibits high thermal stability and mass retention (5% weight loss in an argon atmosphere at 380.6 °C and 75.32% mass retention at 800 °C), and low room temperature thermal conductivity of 0.0551 W m^−1^ K^−1^. The thermal conductivity of PBSA is much lower than that of a silicon-modified polybenzoxazine aerogel at the same mass residual [16].

An additional method for enhancing the LCF/PR mass residual rate and thermal stability is to replace the carbon fiber with inorganic ceramic fibers, including mullite fibers, quartz fibers, glass fibers, and so on [10,25,26]. Compared to carbon and organic fibers, ceramic fibers are more suitable for use in high-temperature aerobic environments due to their resistance to high-temperature oxidation, which can inhibit the scouring of high-temperature aerobic airflow [25,27]. Among them, quartz fiber has received a lot of attention due to its low thermal conductivity, thermal expansion coefficient, high thermal stability and oxidation resistance [4,16]. Furthermore, quartz fibers can be melted at lower temperatures to form a liquid film on the heated surface of the thermal protection material. This hinders the diffusion of high-temperature oxidizing gas streams into the interior of the material, thereby forming a protective layer on the hot surface. Researchers simulated the effect of quartz fiber orientation on the thermal conductivity of ceramic fiber felts [28]. It was found that the best suppression of thermal conduction and radiative heat transfer was achieved when the fibers were oriented and aligned perpendicular to the incident light or temperature gradient. This means that when choosing fibers as the reinforcing phase, a layered structure should be chosen so that the direction of the fiber is as perpendicular as possible to the direction of radiant heat transfer when the material is used at an elevated temperature. The preparation methods of quartz fiber with different diameters are varied, which leads to different fiber lengths. The length of the fiber increases with the diameter of the fibers, the fibers with smaller diameters cannot be prepared into long fibers. Since fiber felt is to be used in the reinforcing phase, this article only focuses on the structure and properties of composites in the z-direction. The intrinsic properties of fibers play a critical role in determining the mechanical properties, and thermal insulation of the final aerogel composites. Unfortunately, important advances in the effect of fiber properties on the performance of lightweight resin-based ablative thermal protection materials have been almost non-existent. Therefore, investigating the effect of fiber properties on the structure and properties of aerogel composites is urgently needed. Generally speaking, the combination of aerogel and fiber is achieved using hydrogen bonding or other weak interactions. When experiencing an extreme aerodynamic thermal environment, it is very easy for the phenomenon of peeling off in aerogel and quartz fibers due to the high-temperature shear of the load, which leads to holes and defects in the thermal protection material, and then threatens the normal flight of the aircraft. The PBS aerogel provided in this article can be chemically reacted with quartz fibers, so that the aerogel can be chemically bonded with the quartz fibers, avoiding the phenomenon of peeling off of the aerogel from the fibers due to the high-temperature shear of the composite material in high-temperature aerobic environments.

Herein, we prepared lightweight multiscale, and high-temperature thermal insulation aerogel composites using a simple copolymerization with ambient pressure drying through the synergistic effect of quartz fiber felts and silica-polybenzoxazine nanoporous aerogels. The influence of fiber diameter on the structure and properties of the aerogel composites was investigated. The specific surface area and average pore diameter of the QF/PBSAs changed with the fiber diameter due to the synergistic effect of the aerogel and the fibers. The mechanical and thermal insulation properties of the QF/PBSAs were significantly influenced by a change in pore structure and the structural characteristics of the fiber felt with different diameters. The results of this study will provide a scientific foundation for the subsequent selection of materials across various applications involving polybenzoxazine aerogel composites.

## 2. Results and Discussion

### 2.1. Influence of Fiber Diameter on Morphology and Textural Properties of QF/PBSAs

**Figure 1:**
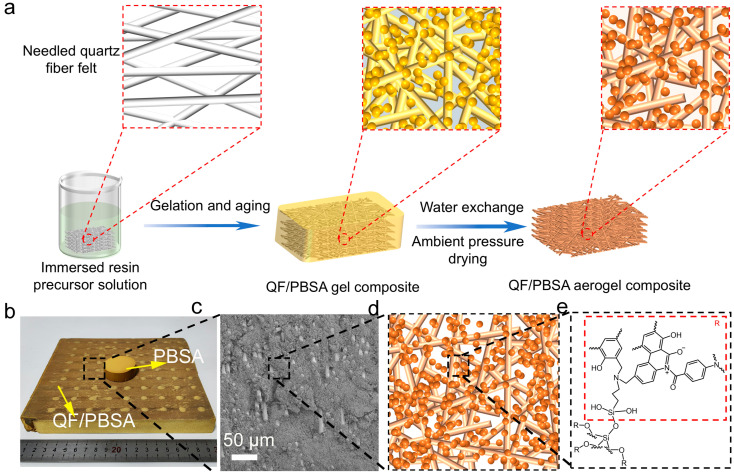
(**a**) Schematic illustration of the synthesized process of the QF/PBSAs. (**b**,**c**) Representative macrophotographs and fracture surface morphology of PBSA and QF/PBSAs. (**d**) Schematic microstructure of QF/PBSAs in xy direction. (**e**) Chemical structure of PBS aerogel.

The preparation process, macroscopic photograph, microscopic morphology, nanoscopic structure, and cross-linking structure of quartz fiber felt-reinforced silica-polybenzoxazine aerogel composites (QF/PBSAs) are shown in Figure 1 and described in detail in Section 4.2.

**Figure 2:**
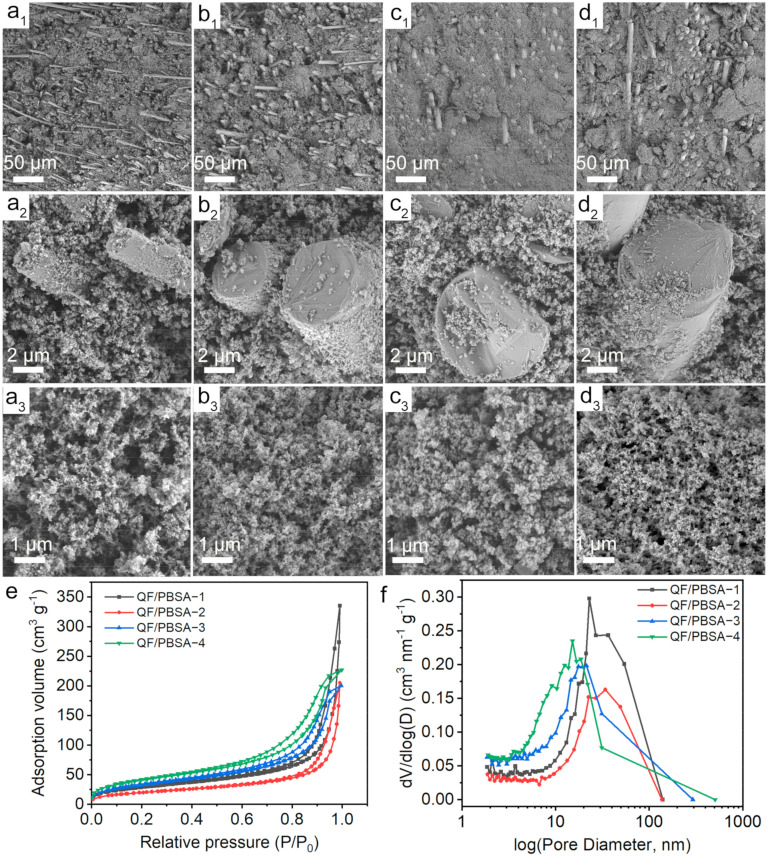
Morphologies of QF/PBSAs. SEM images of: (**a**) QF/PBSA−1; (**b**) QF/PBSA−2; (**c**) QF/PBSA−3; (**d**) QF/PBSA−4; (**e**) N_2_ adsorption and desorption isotherms at 77 K; (**f**) BJH pore diameter distribution curves of QF/PBSAs.

To reveal the influence of fiber diameter on the structure of the QF/PBSAs, a series of characterizations were carried out (Figure 2). SEM images were used to evaluate the morphology of the samples (Figure 2a–d). The morphologies of the QF/PBSAs are similar, indicating that the morphology is not affected by the fiber diameter. Owing to the strong interaction between PBS nanoparticles and QF through the connection of BSi, QFs with different diameters were integrated tightly with PBS aerogel.

Figure 2e,f show N_2_ adsorption and desorption isotherms at 77 K, which exhibit typical type Ⅳ isotherms and H3 hysteresis loops at high relative pressures according to IUPAC classification, indicating mesopores and macropores in the QF/PBSAs [27]. The QF/PBSAs showed obviously decreased N_2_ adsorption with increasing fiber diameter, reflecting a reduced total pore content, which aligns with the pattern of pore structure parameters presented in Table 1. As summarized in Table 1, the mesopore-specific surface area (Smeso) showed a gradual increase with the diameter of the QFs, while the mesopore volume showed opposite trends. In particular, the micropores showed a significantly decreasing trend until they disappeared. This phenomenon occurred because larger fiber diameters led to increased pore size and higher density of the QF/PBSAs, resulting in a denser porous structure. (Figure 2a–d). Furthermore, the robust interaction between PBS nanoparticles and QFs prompted the PBS nanoparticles to grow in conjunction with the fibers, acting as a template, and filling the interstitial spaces between the pores of fibers, thereby forming a PBS aerogel [29]. With an increase in fiber diameter, the specific surface area of the single fiber exhibited a gradual increase, while the connection points between the fibers became fewer. In addition, the pores within the composite were filled with the PBS aerogel, which was formed using SiO_2_ nanoparticles as the template for the porous structure. The formation of larger pores between the fibers resulted in a larger region for the aerogel to be formed, leading to an increased mesoporous structure and an altered pore diameter distribution. All of the above-mentioned issues may be the primary factors influencing the pore structure. As shown in Figure 2f, the pore distribution of the QF/PBSAs displayed broad peaks of a combination of mesopores and macropores concentrated from 10–100 nm to 5–60 nm. The remarkable changes are in good agreement with the analysis of the N_2_ adsorption–desorption behavior. The presence of pores with a diameter of less than 70 nm, favors the limitation of heat transfer within the composite [30].

The pore structure was quantitatively evaluated by total open pore volume and average pore diameter analysis. With an increase in fiber diameter, the overall trend of V_total_, which represents the complete open porosity of the aerogels, slightly increased, and the V_1.7–300nm_/V_total_ ratio decreased, indicating an increase in the number of macropores [31]. Compared to a pure PBS aerogel, the V_1.7–300nm_/V_total_ ratio of the QF/PBSAs decreased, which represents an increase in the number of macropores with the introduction of fibers [32]. The average pore diameter calculated from 4V_total_/S_BET_ is in close agreement with the values obtained from the Hg intrusion porosimetry tests [33]. The analysis of the pore structures in the composites reveals that the majority of the pores are macro–meso-pores, which indicates a hierarchical porous structure. This structure was predicted to confer a low thermal conductivity upon the composites [15].

Overall, all the results clearly revealed that the pore structure of the QF/PBSAs undergoes a transition from a micro-meso-macro-porous structure to a meso-macro-porous structure with increasing fiber diameter, which promotes the generation of a high specific surface area and changes in the pore diameter distribution of the QF/PBSAs.

### 2.2. Influence of Fiber Diameter on the Mechanical Properties of QF/PBSAs

**Figure 3:**
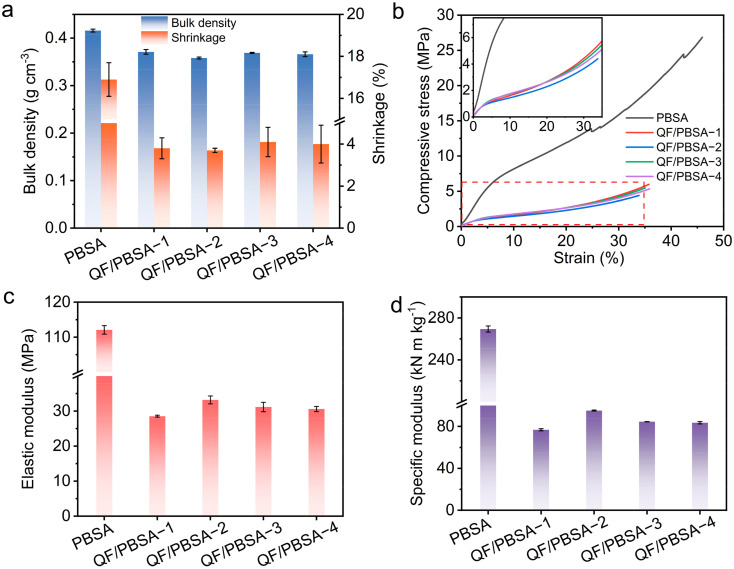
(**a**) Linear shrinkage and density; (**b**) compressive stress−strain curves. Inset: higher magnification image of compressive stress−strain curves; (**c**) elastic modulus; (**d**) specific modulus of PBSA and QF/PBSAs.

PBS aerogels have hierarchical porous structures and hydrogen bond networks formed by the water exchange process, which are the reasons they resist the capillary force of the drying process at an ambient pressure [31]. Nevertheless, despite the successful preparation of the polybenzoxazine aerogel using atmospheric pressure drying, the shrinkage remained considerable (Figure 1b and Figure 3a) and the drying process for larger sizes was highly susceptible to the formation of large cracks in the samples due to capillary forces caused by water evaporation. This rendered the preparation of large-size samples unfeasible. The shrinkage of the composites decreased dramatically after the combination of polybenzoxazine aerogel and quartz fiber due to the strong dimensional shape ability of quartz fiber, and large-size samples were successfully prepared (Figure 1b and Figure 3a). As the fiber diameter increased, the linear shrinkage of the QF/PBSAs gradually increased. While the density showed another fluctuating pattern, initially decreasing, and subsequently increasing (Figure 3a). This phenomenon can be attributed to the combined effects of changes in shrinkage and pore structure resulting from fiber diameter increases.

The mechanical properties of thermal protection materials occupy an important position in their practical application [34,35,36]. The mechanical properties of PBS aerogels are much higher than those of composites due to the strong nanoskeletal structure and higher density of the PBS aerogel, and some pores in the PBS aerogel collapsed during compression (Figure 3b). The compressive stress–strain curves of the QF/PBSAs were similar, displaying three distinct deformation regions: linear elastic, plastic yielding, and densification (Figure 3b). According to the results of morphology and microstructure, the QF/PBSAs possess an abundant hierarchical porous structure with stacked thick-united connection PBS nanoparticles, which means that there is more deformation space inside to avoid damage when compressed [31]. During compression, none of the QF/PBSAs showed brittle fracture (Figure 3b). However, the PBS aerogel showed fracture in the later stages of compression, indicating that the addition of QF increased the toughness of the materials (Figure 3b). The trend of compressive strength with increasing fiber diameter in the linear elastic and plastic-yielding region is as follows: QF/PBSA−4 > QF/PBSA−3 > QF/PBSA−1 > QF/PBSA−2. In the densification region, the trend changes to QF/PBSA−1 > QF/PBSA−3 > QF/PBSA−4 > QF/PBSA−2. This phenomenon may be due to an increase in the fiber diameter, maintaining a constant density, resulting in a reduction in the quantity of connection points with the aerogel composite. The porous structure of aerogel composites provides a deformation space in the linear elastic and plastic-yielding region; however, during densification, more connection points between the PBS aerogel and the QF enhance the compressive strength [37].

The elastic modulus of PBSA, QF/PBSA−1, QF/PBSA−2, QF/PBSA−3, and QF/PBSA−4 were 112.11, 28.52, 33.17, 31.16, and 30.57 MPa, respectively. The corresponding specific modulus were 269.51, 76.89, 92.54, 84.56, and 83.54 kN m kg^−1^, respectively (Figure 3c). PBS aerogels showed much higher elastic modulus and specific modulus than those of QF/PBSAs due to the strong nanoskeletal structure and higher density of the PBS aerogel. The elastic modulus and specification modulus of the QF/PBSAs show a pattern, as the fiber diameter increased, they initially increased, and then slightly decreased, which was related to the change of density and porous structure caused by the fiber diameter. The results revealed a close correlation between the fiber diameter and the mechanical properties of QF/PBSAs, i.e., an increase in the fiber diameter affects the formation of the pore structure and the number of connections between the aerogel and the fiber, which leads to corresponding changes in the mechanical behavior of the aerogel composites at different stages of loading.

### 2.3. Influence of Fiber Diameter on the Thermal Insulation Properties of QF/PBSAs

**Figure 4:**
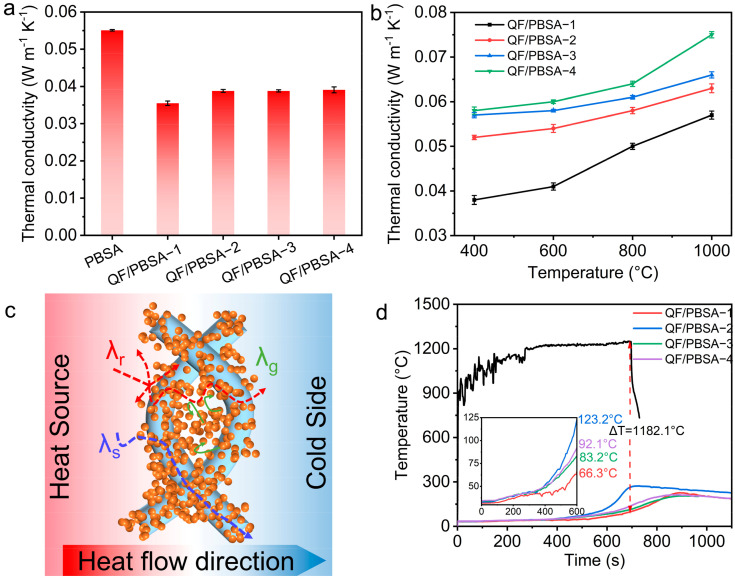
Thermal insulation properties of PBSA and QF/PBSAs: (**a**) thermal conductivities of PBSA and QF/PBSAs at ambient pressure; (**b**) thermal conductivities from 400 to 1000 °C; (**c**) schematic heat transfer mechanism of QF/PBSAs; (**d**) surface and backside temperatures after the butane flame test.

Thermal insulation represents a pivotal performance metric for thermal protection materials, with a strong correlation to the structural characteristic of the material [8,38]. Generally, the lower thermal conductivity of the materials leads to a better property of it in thermal insulation. Theoretically, effective thermal conductivity (λ_total_) can be attributed to gas thermal conductivity (λ_g_), solid thermal conductivity (λ_s_), radiative thermal conductivity (λ_r_) and convective thermal conductivity (λ_c_) [3,39,40]. λ_g_, including the equivalent gas thermal conductivity (λ_g,e_) and the solid-gas coupling thermal conductivity (λ_s–g_), is affected by average pore diameter and porosity [41]. λ_g_ plays an important role in the λ_total_ and increases with the increase in average pore diameter and porosity [41]. At relatively low temperatures, λ_c_ is usually negligible when the pore diameter is less than 1 mm at ambient pressure, so it is not considered in this study [15]. Thus, the thermal conductivity of aerogel composites at room temperature is mainly dependent on the density and pore structure of the materials. The average pore diameter and porosity first increase and then decrease with the increase in fiber diameter (Table 1). λ_g_ is affected by the pore structure of the material, which means that the λ_g_ follows the same trend [41]. The bulk density of the QF/PBSAs showed a tendency to decrease slightly and then increase slightly with the increase in fiber diameter (Figure 3a) combined with the trend of the QF/PBSAs’ specific surface area, which decreases and then increases as the fiber diameter increases (Table 1). λ_total_ exhibits a trend of slowly increasing when the fiber diameter increases, as shown in Figure 4a. For QF/PBSA−2, λ_s_ cannot compensate for the increase in λ_g_ due to the increase in pore diameter and specific surface area, resulting in a higher thermal conductivity for QF/PBSA−2 than for QF/PBSA−1. As the fiber diameter continues to increase, the S_BET_ of the QF/PBSAs increases sharply, the pore diameter decreases significantly, and the porosity remains almost constant, which results in a decrease in the λ_g_. However, compared to QF/PBSA−2, the densities of QF/PBSA−3 and QF/PBSA−4 increased significantly. In this case, λ_g_ can no longer compensate for the increase in λ_s_, thus resulting in an upward trend in the overall thermal conductivity of the composite. These phenomena contributed to the effects of changes in the density and the pore structure of the QF/PBSAs [33,41].

The thermal conductivities of the QF/PBSAs at room temperature were measured using FOX200, which is based on the steady-state method. The thermal conductivities of the QF/PBSAs at high temperatures were tested using the water flow plate method (PBD-12-4Y), which is a non-standard high-temperature thermal conductivity testing method developed based on the steady-state method. Both of these require samples with a diameter of φ180 mm. However, the large shrinkage of the PBS aerogel prevented the successful preparation of large-size samples. The thermal conductivity of the PBS aerogels at room temperature could only be tested using a Hot disk meter (TPS 2500s). Consequently, subsequent tests conducted at elevated temperatures are solely addressed in the context of composite materials.

With an increasing temperature, λ_r_ rises sharply, becoming the dominant heat transfer mode [42,43]. Therefore, effectively inhibiting λ_r_ at high temperatures is crucial for mitigating heat transfer. The heat transfer mechanism of the QF/PBSAs is shown in Figure 4c. The literature indicates that a key method for suppressing high-temperature radiation involves reducing the spectral attenuation coefficient of the aerogel materials [28]. When the temperature changes, the optimal fiber diameter with the highest attenuation coefficient varies at different temperatures due to the different incident wavelengths of the radiation. The optimal diameter of the SiO_2_ fibers gradually decreases with increasing temperature from 9.6 μm at 200 °C to 3.1 μm at 1000 °C [28]. The thermal conductivities of QF/PBSA−1 exhibited a sharply increasing pattern with increasing temperature, from 0.038 W m^−1^ K^−1^ at 400 °C to 0.057 W m^−1^ K^−1^ at 1000 °C, respectively, mainly because λ_r_ increases rapidly with increasing temperature (Figure 4b). The optimum fiber diameter for the highest spectral attenuation coefficient is 3.1 μm at 1000 °C. The spectral attenuation coefficient was decreased with the increase in fiber diameter, leading to a sharp increase in λ_r_. In this case, the λ_s_ of QF/PBSA−2 can no longer compensate for the increase in λ_r_ and λ_g_ compared to QF/PBSA−1, which leads to an upward trend in the λ_total_ of QF/PBSA−2. However, with the increase in fiber diameter (QF/PBSA−3 and QF/PBSA−4), λ_g_ can no longer compensate for the increase in λ_r_ and λ_s_, resulting in a continued increase in the λ_total_ of QF/PBSA−3 and QF/PBSA−4. Among them, the thermal conductivity of QF/PBSA−1 at 400 °C was significantly lower than that of other samples. In this case, the suppression of λ_r_ partially compensates for the decrease in λ_g_ [28].

To simulate the actual service environment, the samples that were 20 mm thick were tested using a 1200 °C butane flame, as shown in Figure 4d. During the test, the QF/PBSAs were exposed to the 1200 °C butane flame, with the backside temperature increasing to 66.3, 123.2, 83.2, and 92.1 °C in 600 s, respectively (Figure 4d). This is distinct from the previous trend of increased thermal conductivity at high temperatures with an increasing fiber diameter. The reasons are as follows: when exposed to an ultrahigh temperature aerobic environment, the surface layer of the QF/PBSAs was pyrolyzed into CO, CO_2_, hydrocarbons, nitrogen hydrocarbons, and other small molecules, which were injected into the boundary layer to form a thermal barrier effect, preventing further heat and O_2_ transfer to the interior and evaporating some pyrolysis gases to carry away heat from the boundary layer. Consequently, higher sample density results in more heat loss through ablation, reducing heat and O_2_ transfer to the interior of the sample. This results in a lower backside temperature for QF/PBSA−1, QF/PBSA−3, and QF/PBSA−4 compared to QF/PBSA−2, even though QF/PBSA−2 has the lowest bulk density and a higher spectral attenuation coefficient. Particularly, QF/PBSA−1 exhibited the lowest backside temperature owing to the low thermal conductivity and spectral attenuation coefficient at high temperatures.

## 3. Conclusions

In this paper, QF/PBSAs were prepared with different fiber diameters using ambient pressure drying and the effects of fiber diameter on the morphologies and pore structures, mechanical properties, and thermal insulation performance at room temperature and elevated temperature were analyzed, respectively. The main conclusions are summarized as follows:

(1) With an increasing fiber diameter, the specific surface area and the mesoporous structure of the composite increased. The reason for the elevated specific surface area and alteration in pore diameter distribution is attributed to the augmented aerogel-filled region between the fiber pores, which is a consequence of the reduction in fiber connection points due to the increase in fiber diameter.

(2) The special stress–strain behavior of the composites in compression is due to the formation of a porous structure and a reduction in the fiber connection points, which are impacted by the increase in fiber diameter.

(3) For the conditions and fibers investigated in this paper, the λ_r_ of the QF/PBSAs can be significantly suppressed at high temperatures as the fiber diameter was 1~3 μm, which apparently reduces the thermal conductivity of the composite at high temperatures. The thermal conductivity of the QF/PBSAs at high temperatures is affected by the combination of density, pore structure, and the λ_r_ of the composites, with λ_r_ playing a dominant role. To simulate the actual application environment, λ_r_, S_meso_, and λ_s_ rapidly increased with increasing fiber diameter. QF/PBSA−1 exhibited the lowest backside temperature due to the lower λ_r_ and λ_s_, and in this case, the suppression of λ_r_ can compensate for the increase in λ_g_ and λ_s_ compared to QF/PBSA−2, QF/PBSA−3, and QF/PBSA−4, which showed lower backside temperature due to their higher bulk densities. At high temperatures, the pyrolysis of the denser PBS aerogel takes more heat away, compensating for the heat transfer due to the increased radiative thermal conductivity.

## 4. Materials and Methods

### 4.1. Materials

DMF-soluble silica sol was prepared by solvent exchange from water-soluble silica sol. Detailed preparation methods for PBO sol and DMF-soluble SiO_2_ sol can be found in our previous work. Quartz fiber felts with different diameters (220 × 220 × 20 mm^3^, natural density of 0.15 g cm^−3^, 1~3, 3~5, 7.5, 8~10 μm) were purchased from Hubei Feilihua Quartz Glass Co., Ltd., Jingmen, Hubei, China. Deionized water (DIW) was used in the experiments.

### 4.2. Preparation of QF/PBSAs

Quartz fiber felts with different diameters were impregnated with proportionally mixed PBO sol and SiO_2_ sol under vacuum for 4 h. The obtained fiber/sol mixture was subjected to gelation, aging, water exchange and ambient pressure drying, then the fiber-reinforced SiO_2_-PBO aerogel composites were obtained. The composites were defined as QF/PBSAx, where x represents the fiber diameter (1, 2, 3, 4 represent fiber diameter of 1~3, 3~5, 7.5, 8~10 μm, respectively).

Figure 1a shows the preparation process of quartz fiber felt-reinforced silica-polybenzoxazine aerogel composites (QF/PBSAs), which is expected to highlight the interaction between quartz fiber (QF) and silica-polybenzoxazine (PBS) precursor during the compound process. The QF used herein has a low density of 0.15 g cm^−3^ with various diameters. First, the QF was immersed in the PBS precursor sol (PBO/SiO_2_/BSi/DMF/EtOH), and vacuum impregnation was used to ensure complete impregnation. Then, the mixture in a sealed container was heated at 80 °C to produce a heterogeneous wet QF/PBS gel composite. To reduce the capillary pressure during water exchange, the wet QF/PBS gel composite was washed with hot water [44,45]. Finally, the QF/PBS hydrogel composite was directly dried to a constant weight in an oven at 50 °C and then step-cured in the procedures of 160 °C 2 h, 180 °C 2 h and 200 °C 2 h [32]. The obtained QF/PBS aerogel composite density was between 0.349 and 0.371 g cm^−3^.

The PBS aerogel was uniformly distributed within the QF and integrated tightly without obvious defects such as cracks or holes (Figure 1b−c). The key to the preparation is to use BSi as the cross-linking agent to cross-link and polymerize the diluent PBO and SiO_2_ precursor into a gel molecular network using the oxazine rings and silicon hydroxyl groups derived from BSi (Figure 1d−e). The results showed that the obtained QF/PBSAs have hierarchical nanoporous structure, which can efficiently resist capillary force-lead pore structure destruction during atmospheric pressure drying owing to the robust QF/PBS hydrogel composite [31]. The preparation process is cost-effective and low pollution, avoiding high-cost and pollution processes such as supercritical drying and an organic solvent exchange.

### 4.3. Characterization

The morphologies of the aerogels were observed by using a field-emission scanning electron microscope (SEM, ZEISS Sigma 300, Jena, Germany) with an accelerating voltage of 3 kV. The specific surface area and the pore diameter distribution of the PBSAs aerogels were determined by using a 3H-2000PM2 analyzer (BeiShiDe Instrument, Beijing, China) and Barrett–Joyner–Halenda (BJH) methods. The bulk densities (*ρ_b_*) were determined by measuring the mass and physical size of the aerogels. The Micromeritics AccuPyc II 1340 instrument was used to obtain skeletal densities (*ρ*_s_) through helium pycnometry. The average pore diameter for samples was measured with Hg-intrusion porosimetry using a Micromeritics Autopore Ⅴ 9600. Fourier transform infrared (FTIR) spectroscopy was conducted by using a Vertex 70 (Bruker, Billerica, MA, USA) instrument, and the wavenumber ranges from 400 cm^−1^ to 4000 cm^−1^. Compressive strength was evaluated with an FL4204GL universal testing machine (Fule Test Technology, Bella Vista Pkwy Warrenville, IL, USA) at a speed of 1 mm min^−1^. The room temperature and high-temperature thermal conductivities of QF/PBSAs were measured using heat flow meter method (FOX200) and water flow plate method (PBD-12-4Y) with a sample dimension of φ180 mm. Thermal conductivity of PBS aerogel at room temperature was measured by using a Hot disk meter (TPS 2500s, Hot disk, Zurich, Switzerland). The high-temperature thermal insulation property of QF/PBSAs was measured by using a 1200 °C butane flame, and the surface and backside temperatures of the sample were monitored using thermocouples.

## Figures and Tables

**Table 1:** The pore structure parameters of PBSA and QF/PBSAs.

Samples	S_BET_ (m^2^ g^−1^)	S_meso_ (m^2^ g^−1^)	S_mic_ (m^2^ g^−1^)	V_total_ (cm^3^ g^−1^)	V_1.7~300nm_ (cm^3^ g^−1^)	V_mic_ (cm^3^ g^−1^)	Porosity (%)	Average Pore Diameter (nm)	
								4V_total_/S_BET_	from Hg Intrusion
PBSA	87.74	78.39	9.35	1.82	0.5343	0.0039	75.8	83.1	42.2
QF/PBSA−1	104.45	88.28	16.17	2.17	0.5396	0.0078	80.4	80.9	107.3
QF/PBSA−2	71.52	60.58	10.94	2.28	0.3314	0.0057	81.1	127.3	180.7
QF/PBSA−3	120.47	120.47	0	2.19	0.3332	0	80.5	72.6	103.2
QF/PBSA−4	147.19	147.19	0	2.21	0.3770	0	80.7	60.1	108.8

S_BET_: BET surface area; S_meso_: BJH desorption cumulative surface area; S_mic_: Micropore surface area; V_total_ = (1/ρ_b_) − (1/ρ_s_); V_1.7~300nm_: BJH desorption cumulative pore volume; V_mic_: Micropore volume; Porosity = (1 − ρ_b_/ρ_s_) × 100, ρ_b:_ bulk density, ρ_s_: skeletal density.

## Data Availability

The data presented in this study are available on request from the corresponding author.
